# Time-domain Formulation of a Multi-layer Plane Circuit Coupled with Lumped-parameter Circuits using Maxwell Equations

**DOI:** 10.1038/s41598-019-53288-x

**Published:** 2019-11-29

**Authors:** Souma Jinno, Shuji Kitora, Hiroshi Toki, Masayuki Abe

**Affiliations:** Graduate School of Engineering Science, Osaka University, Toyonaka, Osaka 560-8531 Japan

**Keywords:** Applied mathematics, Electrical and electronic engineering

## Abstract

We calculate electromagnetic phenomena in the multi-layer plane circuit starting from the Maxwell equations. We present a numerical method of potential and current density in two-dimensional conductors, where their time developments are treated as phenomena of wave propagation. We treat the plane conductors by dividing them into small finite-volume elements, similar to the case of the partial element equivalent circuit method, and the transport equations are then solved by the finite-difference time-domain method. Furthermore, we develop a calculation method for the boundary in a multi-layer plane by applying the method we have used in multi-transmission lines. We formulate the boundary conditions of a multi-layer plane coupled with lumped-parameter circuits and introduce an algorithm to reduce calculation costs that are largely associated with the two-dimensional extension from the multi-transmission-line case. We perform calculations of the wave propagation of potential, current density, and charge density in the time domain for a simple plane circuit. These calculations are presented as supplementary materials of the present paper.

## Introduction

Electromagnetic noise is troublesome because it is difficult to understand the fundamental phenomena associated with noise. In recent years, circuit design that reduces the influence of electromagnetic noise has become increasingly important for reliable performance with the trend toward high-frequency and low-voltage electronics^1^. This trend is likely to result in electromagnetic noise, causing the malfunction of electric devices, and can be a major bottleneck in future circuit design^2^. There are many symptomatic treatments, such as filters, to suppress the electromagnetic noise^3,4^. However, these treatments require the experience of skilled engineers because the causes of electromagnetic noise phenomena are not clearly understood and cannot be described quantitatively using equivalent circuit modeling and impedance analysis in the frequency domain^5,6^. A fundamental understanding of the mechanisms of electromagnetic noise is needed for noiseless circuit design.

In previous studies, we elucidated the mechanism of electromagnetic noise phenomena in line circuit systems using the multi-conductor transmission line (MTL) theory derived from the Maxwell equations^7^. We calculated common-mode noise for a configuration of three-transmission-line theory and developed a noiseless circuit design^8,9^. We have also proposed a numerical calculation algorithm for hybrid systems, where the distributed- and lumped-parameter circuits are connected^10,11^.

For further analysis of electromagnetic noise in an actual circuit, such as a printed circuit board (PCB), we have extended the MTL theory to two- and three-dimensional conductors and formulated transport equations of potential and current. In the numerical calculation, we realized calculation of a multi-layer plane (MLP) circuit with a discontinuous ground using the finite-difference time-domain (FDTD) method^12^. Although we applied our algorithm to an MLP circuit to connect lumped-parameter circuits at boundaries, there are still major problems to be solved. First, we have to consider the directions of current according to the locations of the boundaries in the MLP. Second, the number of connection points of the lumped-parameter circuit greatly increases as compared to the case of the MTL theory. For these reasons, applying the previous calculation algorithm to arbitrary shapes of MLPs is difficult due to complicated boundary conditions and the associated heavy computer burden.

In the present paper, we would like to extend the MTL theory to the case of two- and three-dimensional conductors. To this end, we derive the transport equations in the form of finite-volume elements of potential and current density from the Maxwell equations. Using these equations, we are able to visualize the transitional phenomena as a function of time, which is important in order to understand the electromagnetic noise phenomena. In addition, we would like to resolve problems of complex calculations at boundaries of MLPs. For this purpose, we formulate the boundary equations to consider any direction of current at the boundaries. We use Kirchhoff’s current law (KCL) to consider the connective relation between lumped- and distributed-parameter circuits. We also use Kirchhoff’s voltage law (KVL) and branch constitutive equation (BCE) to consider the lumped-parameter circuit conditions.

**Figure 1:**
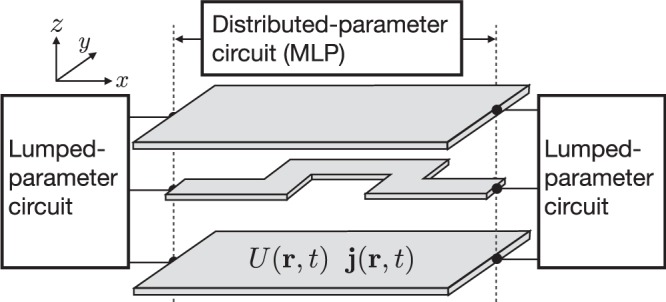
Example of a two-dimensional multi-conductor plane circuit system described in the present paper. We regard MLP circuits as distributed-parameter circuits. Lumped-parameter circuits, such as voltage source, resistance, capacitance, or inductance circuits, are connected by distributed-parameter circuits at the boundaries, which provide the boundary conditions. The potential *U* and current density ***j*** depend on the position **r** and time *t*.

In the present paper, a calculation algorithm for a two-dimensional conductor is presented in order to clarify the mechanism of electromagnetic noise occurring in the MLP circuit. We assume that two- or three-dimensional MLP circuit systems are connected with lumped-parameter circuits at the boundaries (an example is shown in Fig. 1). The proposed method provides information of transient phenomena of the potential and current density in the planar circuit system. In Section 2, two- and three-dimensional telegraphic equations expressed in terms of potential and current density are derived. Then, coupled differential equations are expressed in the form of discretized finite-volume elements for numerical calculation. In Section 3, we introduce the numerical method at the boundary. In Section 4, we describe how to reduce boundary calculation costs by considering the fact that most of the boundary of the MLP is open, by which the calculation cost of the boundary conditions can be significantly reduced.

## Derivation of Coupled Difference Equations of Potential and Current Density in MLP Circuits

In previous studies, we derived equations of potential and current in a multi-transmission-line (MTL) system from the Maxwell equations^7^. We have reported discretized equations in an MLP circuit system and compared the results of simulations and experiments^12^. In the present paper, we discuss the derivation of discretized equations in detail in order to further understand the calculations. We start with the retarded scalar $U({\bf{r}},t)$ and vector ***A***$({\bf{r}},t)$ potentials obtained by applying the Lorenz gauge from the Maxwell equations.

1 $$U({\bf{r}},t)=\frac{1}{4\pi \varepsilon }\,\int \,{\rm{d}}{\bf{r}}{\rm{^{\prime} }}\frac{q({\bf{r}}{\rm{^{\prime} }},t-\frac{|{\bf{r}}-{\bf{r}}{\rm{^{\prime} }}|}{c})}{|{\bf{r}}-{\bf{r}}{\rm{^{\prime} }}|},$$

2 $${\boldsymbol{A}}({\bf{r}},t)=\frac{\mu }{4\pi }\,\int \,{\rm{d}}{\bf{r}}{\rm{^{\prime} }}\frac{{\boldsymbol{j}}({\bf{r}}{\rm{^{\prime} }},t-\frac{|{\bf{r}}-{\bf{r}}{\rm{^{\prime} }}|}{c})}{|{\bf{r}}-{\bf{r}}{\rm{^{\prime} }}|},$$

where $q({\bf{r}},t)$ and ***j***$({\bf{r}},t)$ are the charge and current densities in the conductors. We assume that, in addition to $U({\bf{r}},t)$ and ***A***$({\bf{r}},t)$, both Ohm’s law and charge conservation hold in the planar conductors.

3 $$-\nabla U({\bf{r}},t)-\frac{\partial \,{\boldsymbol{A}}({\bf{r}},t)}{\partial t}=\rho {\boldsymbol{j}}({\bf{r}},t),$$

4 $$\frac{\partial }{\partial t}q({\bf{r}},t)-\nabla \cdot {\boldsymbol{j}}({\bf{r}},t)=0.$$

Here, $\rho$ denotes the resistance in the plane conductors.

**Figure 2:**
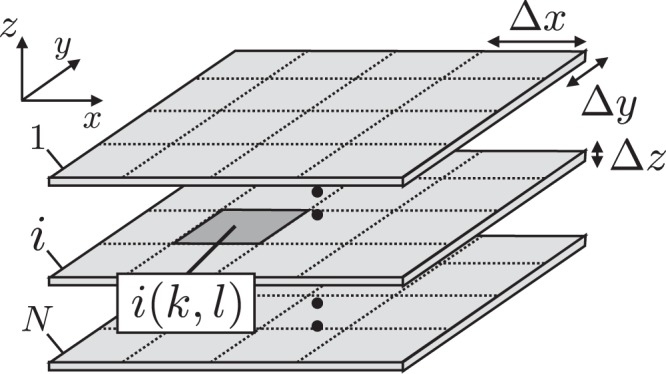
We introduce small finite-volume elements in the multi-plane conductors. The $(x,y)$ position of the *i*-th plane is discretized as $i(k,l)$, where *k* and *l* correspond to numbers in the *x* and *y* directions, respectively. The volume of each small element is $V=\Delta x\Delta y\Delta z$, where Δ*z* is the thickness of the plane and is considered to be very thin.

These four equations satisfy the necessary and sufficient conditions for solving the scalar potential, vector potential, charge density, and current density in conductors. Here, we realize a numerical instability in the presence of the delay time in Eqs.. (1) and (2). This stability problem is a large challenge and a long-standing problem for circuit theories such as the partial element equivalent circuit (PEEC) method^13^. At present, we avoid this numerical difficulty by neglecting the delay time in the coupled differential equations^14^. For numerical calculation, we use finite-volume elements by taking an average in the small finite volume. In the present paper, we assume a multi-layer planar conductor circuit, as shown in Fig. 2, and we use a cubic finite-volume element to divide the conductor planes into finite-volume elements. We refer to one of the divided elements as cell $i(k,l)$, where *i* is a conductor plane, *k* is the *x* coordinate, *l* is the *y* coordinate, and the mesh dimensions are Δ*x* and Δ*y*. Here, we consider the distribution in two directions (*x* and *y*) because the thickness of the plane conductor, denoted Δ*z*, is sufficiently thin compared with Δ*x* and Δ*y*. The volume of cell $i(k,l)$ is represented by ${V}_{i(k,l)}=\Delta x\Delta y\Delta z$. We can express all variables using quantities of cell elements as:

5 $${U}_{i(k,l)}(t)=\frac{1}{{V}_{i(k,l)}}\,{\int }_{{V}_{i(k,l)}}\,U({\bf{r}},t){\rm{d}}{\bf{r}},\,{{\boldsymbol{A}}}_{i(k,l)}(t)=\frac{1}{{V}_{i(k,l)}}\,{\int }_{{V}_{i(k,l)}}\,{\boldsymbol{A}}({\bf{r}},t){\rm{d}}{\bf{r}},$$

6 $${q}_{i(k,l)}(t)=\frac{1}{{V}_{i(k,l)}}\,{\int }_{{V}_{i(k,l)}}\,q({\bf{r}},t){\rm{d}}{\bf{r}},\,{{\boldsymbol{j}}}_{i(k,l)}(t)=\frac{1}{{V}_{i(k,l)}}\,{\int }_{{V}_{i(k,l)}}\,{\boldsymbol{j}}({\bf{r}},t){\rm{d}}{\bf{r}}.$$

We apply the averaging procedure to cell $i(k,l)$ for Eqs. (1) and (2):

7 $${U}_{i(k,l)}(t)=\sum _{i^{\prime} ,k^{\prime} ,l^{\prime} }\,{P}_{i(k,l)i^{\prime} (k^{\prime} ,l^{\prime} )}{q}_{i^{\prime} (k^{\prime} ,l^{\prime} )}(t).$$

8 $${{\boldsymbol{A}}}_{i(k,l)}(t)=\sum _{i^{\prime} ,k^{\prime} ,l^{\prime} }\,{L}_{i(k,l)i^{\prime} (k^{\prime} ,l^{\prime} )}{{\boldsymbol{j}}}_{i^{\prime} (k^{\prime} ,l^{\prime} )}(t).$$

Equations (1) and (2) can be expressed as the sum of cell elements. Here, *P* and *L* represent the local potential coefficient and the local inductance coefficient of the conductors:

9 $${P}_{i(k,l)i^{\prime} (k^{\prime} ,l^{\prime} )}=\frac{1}{4\pi \varepsilon }\frac{1}{{V}_{i(k,l)}}\,{\int }_{{V}_{i(k,l)}}\,{\int }_{{V}_{i^{\prime} (k^{\prime} ,l^{\prime} )}}\,\frac{1}{|{\bf{r}}-{\bf{r}}^{\prime} |}{\rm{d}}{\bf{r}}{\rm{d}}{\bf{r}}^{\prime} ,$$

10 $${L}_{i(k,l)i^{\prime} (k^{\prime} ,l^{\prime} )}=\frac{\mu }{4\pi }\frac{1}{{V}_{i(k,l)}}\,{\int }_{{V}_{i(k,l)}}\,{\int }_{{V}_{i^{\prime} (k^{\prime} ,l^{\prime} )}}\,\frac{1}{|{\bf{r}}-{\bf{r}}^{\prime} |}{\rm{d}}{\bf{r}}{\rm{d}}{\bf{r}}^{\prime} .$$

The local coefficients *P* and *L* have two sets of subscripts, which represent the relation between cells $i(k,l)$ and $i^{\prime} (k^{\prime} ,l^{\prime} )$. Next, we derive spatial difference equations by taking the average of Eqs. (3) and (4) in cell $i(k,l)$. Here, Eq. (3) includes the spatial differentiation, which is the inclination in the *x*, *y*, and *z* directions in cell $i(k,l)$. For instance, spatial differentiation of any function $f(x,y,z)$ in the *x* direction can be converted into the cell spatial difference using the following relation:

11 $$\begin{array}{c}\frac{1}{{V}_{i(k,l)}}\,{\int }_{{V}_{i(k,l)}}\,\frac{{\rm{\partial }}}{{\rm{\partial }}x}f(x,y,z){\rm{d}}{\bf{r}}\\ \,=\,\frac{1}{\Delta x\Delta y\Delta z}{\int }_{(k-1/2)\Delta x}^{(k+1/2)\Delta x}\,dx\,{\int }_{(l-1/2)\Delta y}^{(l+1/2)\Delta y}\,dy\Delta z\frac{[f(x+\frac{\Delta x}{2},y)-f(x-\frac{\Delta x}{2},y)]}{\Delta x}\\ \,=\,\frac{{f}_{i(k+\frac{1}{2},l)}-{f}_{i(k-\frac{1}{2},l)}}{\Delta x}.\end{array}$$

We express spatial differentiation with respect to *x* as a central difference shifted from *x* by half a cell, and take the average in the *x* direction. The average spatial differentiation in cell $i(k,l)$ is converted into the cell spatial difference between cells, which is positively or negatively shifted by half a cell in the *x* direction from cell $i(k,l)$. Furthermore, the divergence of the space is included in Eq. (4) and can be expressed using the inner product:

12 $$\begin{array}{c}\frac{1}{{V}_{i(k,l)}}\,{\int }_{{V}_{i(k,l)}}\,{\rm{\nabla }}\cdot {\bf{f}}(x,y,z)\,{\rm{d}}{\bf{r}}\\ \,=\,\frac{1}{{V}_{i(k,l)}}\,\,{\int }_{{V}_{i(k,l)}}\,[\frac{{\rm{\partial }}}{{\rm{\partial }}x}{f}_{x}(x,y,z)+\frac{{\rm{\partial }}}{{\rm{\partial }}y}{f}_{y}(x,y,z)+\frac{{\rm{\partial }}}{{\rm{\partial }}z}{f}_{z}(x,y,z)]{\rm{d}}{\bf{r}}\\ \,=\,\frac{{f}_{xi(k+\frac{1}{2},l)}-{f}_{xi(k-\frac{1}{2},l)}}{\Delta x}+\frac{{f}_{yi(k,l+\frac{1}{2})}-{f}_{yi(k,l-\frac{1}{2})}}{\Delta y}.\end{array}$$

Here, we do not consider the thin *z* direction of a rectangular cell for simplicity. The average divergence in cell $i(k,l)$ is converted into the sum of the spatial difference in each direction. Finally, Ohm’s law (3) and the continuous Eq. (4) can be given in terms of the cell spatial differences:

13 $$\frac{{U}_{i(k+1,l)}(t)-{U}_{i(k,l)}(t)}{\Delta x}=-\,\sum _{i^{\prime} ,k^{\prime} ,l^{\prime} }\,{L}_{i(k,l)i^{\prime} (k^{\prime} ,l^{\prime} )}\frac{\partial {j}_{xi^{\prime} (k^{\prime} +\frac{1}{2},l^{\prime} )}(t)}{\partial t}-{\rho }_{i}{j}_{xi(k+\frac{1}{2},l)}(t),$$

14 $$\frac{{U}_{i(k,l+1)}(t)-{U}_{i(k,l)}(t)}{\Delta y}=-\,\sum _{i^{\prime} ,k^{\prime} ,l^{\prime} }\,{L}_{i(k,l)i^{\prime} (k^{\prime} ,l^{\prime} )}\frac{\partial {j}_{yi^{\prime} (k^{\prime} ,l^{\prime} +\frac{1}{2})}(t)}{\partial t}-{\rho }_{i}{j}_{yi(k,l+\frac{1}{2})}(t),$$

15 $$\frac{\partial }{\partial t}{q}_{i(k,l)}(t)-\frac{{j}_{xi(k+\frac{1}{2},l)}-{j}_{xi(k-\frac{1}{2},l)}}{\Delta x}-\frac{{j}_{yi(k,l+\frac{1}{2})}-{j}_{yi(k,l-\frac{1}{2})}}{\Delta y}=0.$$

Here, Eqs. (13) and (14) express the vector components of Eq. (3). The difference of the half integer of space between the potential and the current densities comes from Eqs. (11) and (12). We used the scalar potential and the current density as variables in circuit theory and eliminated the vector potential and charge density cell elements from Eqs. (7), (8), (13), (14) and (15). Finally, the transport equations are expressed in the form of recurrence formulas by discretizing the time direction:

16 $$\begin{array}{rcl}\frac{{U}_{i(k,l)}^{m+1}-{U}_{i(k,l)}^{m}}{\Delta t} & = & -\,\mathop{\sum }\limits_{i^{\prime} ,k^{\prime} ,l^{\prime} }^{N,{N}_{x},{N}_{y}}\,{P}_{i(k,l)i^{\prime} (k^{\prime} ,l^{\prime} )}\frac{{j}_{xi^{\prime} (k^{\prime} +\frac{1}{2},l^{\prime} )}^{m+\frac{1}{2}}-{j}_{xi^{\prime} (k^{\prime} -\frac{1}{2},l^{\prime} )}^{m+\frac{1}{2}}}{\Delta x}\\  &  & -\,\mathop{\sum }\limits_{i^{\prime} ,k^{\prime} ,l^{\prime} }^{N,{N}_{x},{N}_{y}}\,{P}_{i(k,l)i^{\prime} (k^{\prime} ,l^{\prime} )}\frac{{j}_{yi^{\prime} (k^{\prime} ,l^{\prime} +\frac{1}{2})}^{m+\frac{1}{2}}-{j}_{yi^{\prime} (k^{\prime} ,l^{\prime} -\frac{1}{2})}^{m+\frac{1}{2}}}{\Delta y},\end{array}$$

17 $$\begin{array}{rcl}\frac{{U}_{i(k+1,l)}^{m+1}-{U}_{i(k,l)}^{m+1}}{\Delta x} & = & -\,\mathop{\sum }\limits_{i^{\prime} ,k^{\prime} ,l^{\prime} }^{N,{N}_{x},{N}_{y}}\,{L}_{i(k+\frac{1}{2},l)i^{\prime} (k^{\prime} +\frac{1}{2},l^{\prime} )}\frac{{j}_{xi^{\prime} (k^{\prime} +\frac{1}{2},l^{\prime} )}^{m+\frac{3}{2}}-{j}_{xi^{\prime} (k^{\prime} +\frac{1}{2},l^{\prime} )}^{m+\frac{1}{2}}}{\Delta t}\\  &  & -\,{\rho }_{i(k+\frac{1}{2},l)}\frac{{j}_{xi(k+\frac{1}{2},l)}^{m+\frac{3}{2}}+{j}_{xi(k+\frac{1}{2},l)}^{m+\frac{1}{2}}}{2},\end{array}$$

18 $$\begin{array}{rcl}\frac{{U}_{i(k,l+1)}^{m+1}-{U}_{i(k,l)}^{m+1}}{\Delta y} & = & -\,\mathop{\sum }\limits_{i^{\prime} ,k^{\prime} ,l^{\prime} }^{N,{N}_{x},{N}_{y}}\,{L}_{i(k,l+\frac{1}{2})i^{\prime} (k^{\prime} ,l^{\prime} +\frac{1}{2})}\frac{{j}_{yi^{\prime} (k^{\prime} ,l^{\prime} +\frac{1}{2})}^{m+\frac{3}{2}}-{j}_{yi^{\prime} (k^{\prime} ,l^{\prime} +\frac{1}{2})}^{m+\frac{1}{2}}}{\Delta t}\\  &  & -\,{\rho }_{i(k,l+\frac{1}{2})}\frac{{j}_{yi(k,l+\frac{1}{2})}^{m+\frac{3}{2}}+{j}_{yi(k,l+\frac{1}{2})}^{m+\frac{1}{2}}}{2}.\end{array}$$

**Figure 3:**
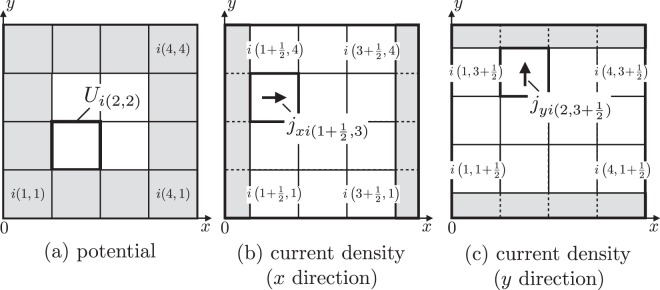
(**a**) Cell element ${U}_{i(k,l)}^{m+1}$ for potential at the *x*, *y* position in the *i*-th plane. In this example, discretized numbers in the *x* and *y* directions are set to ${N}_{x}={N}_{y}=4$. (**b**) Cell element ${j}_{xi(k+1/2,l)}^{m+1}$ for current density in the *x* direction. The flow direction is from left to right. (**c**) Cell element ${j}_{yi(k,l+1/2)}^{m+1}$ for current density in the *y* direction in the *i*-th plane. The flow direction is from below to above. The edges of both directions are discretized with a half width of Δ*x* or Δ*y*, the cells of which are gray and are placed at the boundaries of MLP.

These equations are difference equations for the potential and current density in space and time. We can obtain the potential in the future time from Eq. (16), and the values of current density in the *x* and *y* directions are obtained from Eqs. (17) and (18), respectively. Moreover, if the current densities can be calculated, we can also quantify the vector potential and charge density using Eqs. (8) and (15), respectively. The suffix *m* at the upper right represents the discretized time. The difference of the half integer of time between the potential and current densities comes from the FDTD method. Here, *N* represents the number of plane, and *N*_*x*_ and *N*_*y*_ represent the numbers of divisions in the *x* and *y* directions, respectively. The calculation of boundaries expressed by gray cells in Fig. 3 is explained in the next section.

## Method of Boundary Calculation of MLP with a Lumped-Parameter Circuit

**Figure 4:**
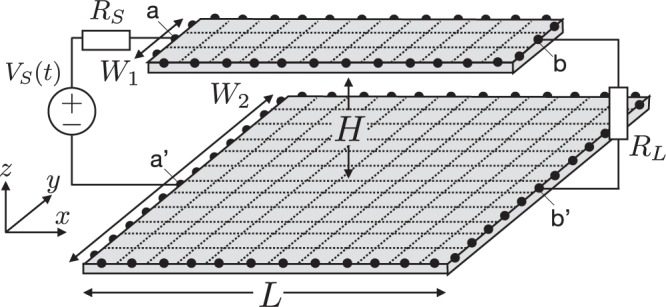
Connection between the lumped- and distributed-parameter circuits at boundaries. In order to use KCL, we assume that the cell potential is equivalent to the node potential and that the cell current is equivalent to the branch current. In the Supplementary information, we present the time variations of potentials and charge densities as numerical calculation results (see Supplementary Information, Movies 1 and 2). The upper plane has a narrower width of *W*_1_, and the bottom plane has a wider width of *W*_2_. The length of both planes is *L*, and the distance between them is *H*. The independent voltage source *V*_*S*_, which has internal resistance *R*_*S*_, is connected at the left side (a-a’), and approximate impedance matching is realized using an appropriate value of *R*_*L*_ at the right side (b-b’).

In this section, we explain the method of connecting the two-dimensional distributed-parameter circuit and the lumped-parameter circuit at any boundary position. We use the node potential and branch current in the lumped-parameter circuit and the cell potential and cell current density in the distributed-parameter circuit as unknown variables. In order to treat these circuits, we use KVL and the BCE in the lumped-parameter circuit and Eqs. (16), (17) and (18), which are cell constitutive equations (CCE), in the distributed-parameter circuit. Assuming that the node potential is equivalent to the cell potential and branch current is equivalent to the cell current, we can express current conservation by KCL, which is the connection relationship between the lumped- and distributed-parameter circuits, as shown in Fig. 4.

We formulated the time-domain incident-potential equation (IPE), which is a coupled equation of a lumped-parameter circuit and an MTL, in a previous study^11^. As for the lumped-parameter circuit, we can use time-domain IPE by using the incidence matrix of directed graph **A** and time-domain impedance matrix **Z**:

19 $$(\begin{array}{cc}{{\bf{A}}}_{l}^{T} & -{{\bf{Z}}}_{l}\\ {\bf{0}} & {{\bf{A}}}_{l}\end{array})(\begin{array}{c}{{\bf{U}}}_{l}^{m+1}\\ {{\bf{I}}}_{l}^{m+1}\end{array})=(\begin{array}{cc}-\epsilon {{\bf{A}}}_{l}^{T} & \delta {{\bf{Z}}}_{l}\\ {\bf{0}} & {{\bf{A}}}_{l}\end{array})(\begin{array}{c}{{\bf{U}}}_{l}^{m}\\ {{\bf{I}}}_{l}^{m}\end{array})+(\begin{array}{c}{{\bf{V}}}_{s}^{m+1}+{{\bf{V}}}_{s}^{m}\\ -{{\bf{A}}}_{s}{{\bf{I}}}_{s}^{m+1}\end{array})$$

Here, the upper right subscript *m* of **U** and **I** refers to discretized time. Unknown variables at a future time are moved to the left-hand side to solve the IPE. Here, **A**_*l*_ is an incidence matrix of size $p\times q$, where *p* is associated with the number of nodes, and *q* is the number of branches. In addition, **A**_*l*_ can express the relationship of connections between nodes and branches, and its entries are 0, 1, or −1. If the component of the *α*-th row and the *β*-th column is 1, then the *α*-th node is connected to the *β*-th branch, and the current direction is outward from the node. If the component of the *α*-th row and the *β*-th column is −1, then the current direction is inward to the node. If the component of the *α*-th row and the *β*-th column is 0, then the nodes and branches are not connected. In addition, **Z**_*l*_ is a time-domain impedance matrix of size $p\times p$, which has diagonal elements depending on *β*-th lumped-parameter circuit elements. From a previous study^10,11^, the time-domain impedance elements are derived as follows. The time-domain impedance elements for the resistor, capacitor, inductor, and independent voltage source are *R*, $\Delta t/(2C)$, $2L/(\Delta t)$, and 0, respectively. Vector **V**_*s*_ is for an independent voltage source, and vector **I**_*s*_ is for an independent current source. Then, $\epsilon$ and *δ* are $p\times p$ diagonal matrices, which are used to change signs depending on the *β*-th lumped-parameter circuit elements:

20 $${\epsilon }_{\beta \beta }=\{\begin{array}{cc}-1 & {\rm{f}}{\rm{o}}{\rm{r}}\,{\rm{c}}{\rm{a}}{\rm{p}}{\rm{a}}{\rm{c}}{\rm{i}}{\rm{t}}{\rm{a}}{\rm{n}}{\rm{c}}{\rm{e}}\\ 1 & {\rm{f}}{\rm{o}}{\rm{r}}\,{\rm{o}}{\rm{t}}{\rm{h}}{\rm{e}}{\rm{r}}\,{\rm{e}}{\rm{l}}{\rm{e}}{\rm{m}}{\rm{e}}{\rm{n}}{\rm{t}}{\rm{s}}\end{array},\,{\delta }_{\beta \beta }=\{\begin{array}{cc}-1 & {\rm{f}}{\rm{o}}{\rm{r}}\,{\rm{i}}{\rm{n}}{\rm{d}}{\rm{u}}{\rm{c}}{\rm{t}}{\rm{a}}{\rm{n}}{\rm{c}}{\rm{e}}\\ 1 & {\rm{f}}{\rm{o}}{\rm{r}}\,{\rm{o}}{\rm{t}}{\rm{h}}{\rm{e}}{\rm{r}}\,{\rm{e}}{\rm{l}}{\rm{e}}{\rm{m}}{\rm{e}}{\rm{n}}{\rm{t}}{\rm{s}}\end{array}.$$

**Figure 5:**
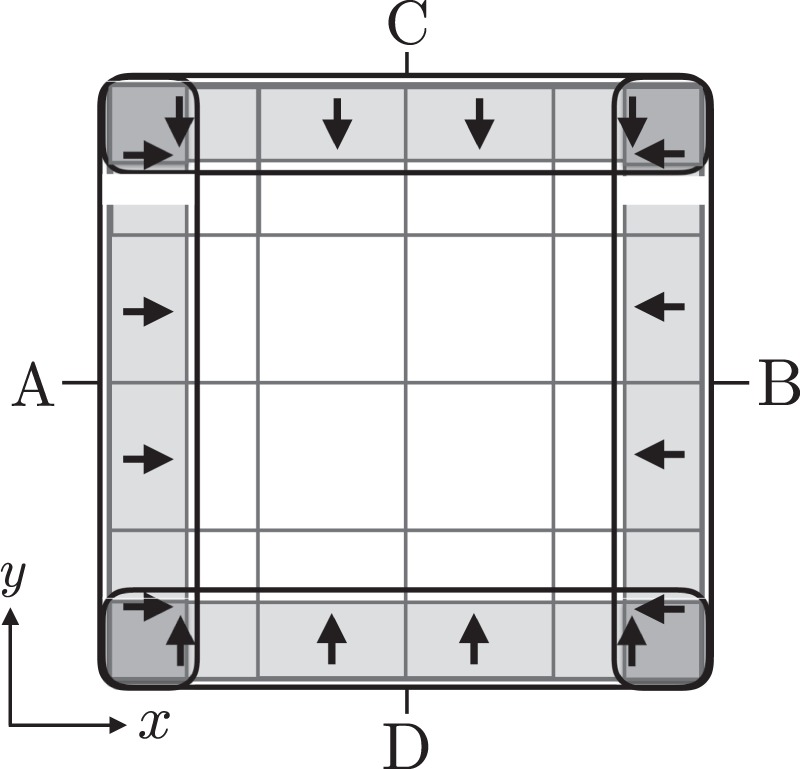
Cell currents flow in the potential cell at the boundaries indicated as A through D. The sign of the equation in the distributed-parameter circuit changes according to the direction of the current at each boundary to be considered in the boundary equations.

Next, we derive the time-domain IPE in the same form as Eq. (19) for the distributed-parameter circuit. We consider boundary equations of lumped-parameter circuits and the MLP circuit at boundaries A through D shown in Fig. 5. Since the size of the current density cell is halved at these boundaries and results in a complicated numerical calculation of the impedance matrix, we replace the boundary current density with the time average of the current density flowing in a potential cell $i(k,l)$ at the boundary, which is expressed as follows:

21 $$\begin{array}{rcl}{j}_{xi(k\pm \frac{1}{2},l)}^{m+\frac{1}{2}} & = & \frac{{j}_{xi(k,l)}^{m+1}+{j}_{xi(k,l)}^{m}}{2},\\ {j}_{yi(k,l\pm \frac{1}{2})}^{m+\frac{1}{2}} & = & \frac{{j}_{yi(k,l)}^{m+1}+{j}_{yi(k,l)}^{m}}{2}.\end{array}$$

This replacement of current densities (21) at the boundaries applies to $k-1/2$ with $k=1$ (Boundary A) and $k+\mathrm{1/2}$ with $k={N}_{x}$ (Boundary B) for *j*_*x*_ and $l-1/2$ with $l=1$ (Boundary D) and $l+1/2$ with $l={N}_{y}$ (Boundary C) for *j*_*y*_.

We use the recurrence formula of the potential in Eq. (16) as a CCE for the boundary equation. Unknown variables in the distributed-parameter circuit are the boundary potential and the current density flowing in the potential cell and are extracted from the summation in Eq. (16), which is brought to the left-hand side:

22 $$\begin{array}{c}{U}_{i(k,l)}^{m+1}-\mathop{\sum }\limits_{{i}^{{\rm{^{\prime} }}},k{\rm{^{\prime} }},l{\rm{^{\prime} }}}^{{\rm{B}}{\rm{o}}{\rm{u}}{\rm{n}}{\rm{d}}{\rm{a}}{\rm{r}}{\rm{y}}}\,\mathop{\sum }\limits_{\alpha }^{x,y}\,{\gamma }_{\alpha i{\rm{^{\prime} }}(k{\rm{^{\prime} }},l{\rm{^{\prime} }})}\frac{\Delta t}{\Delta \alpha }{P}_{i(k,l)i{\rm{^{\prime} }}(k{\rm{^{\prime} }},l{\rm{^{\prime} }})}{j}_{\alpha i{\rm{^{\prime} }}(k{\rm{^{\prime} }},l{\rm{^{\prime} }})}^{m+1}\\ \begin{array}{ccc} & = & {U}_{i(k,l)}^{m}+\mathop{\sum }\limits_{i{\rm{^{\prime} }},k{\rm{^{\prime} }},l{\rm{^{\prime} }}}^{{\rm{B}}{\rm{o}}{\rm{u}}{\rm{n}}{\rm{d}}{\rm{a}}{\rm{r}}{\rm{y}}}\,\mathop{\sum }\limits_{\alpha }^{x,y}\,{\gamma }_{\alpha i{\rm{^{\prime} }}(k{\rm{^{\prime} }},l{\rm{^{\prime} }})}\frac{\Delta t}{\Delta \alpha }{P}_{i(k,l)i{\rm{^{\prime} }}(k{\rm{^{\prime} }},l{\rm{^{\prime} }})}{j}_{\alpha i{\rm{^{\prime} }}(k{\rm{^{\prime} }},l{\rm{^{\prime} }})}^{m}\\  &  & -\,\mathop{\sum }\limits_{i{\rm{^{\prime} }},k{\rm{^{\prime} }},l{\rm{^{\prime} }}}^{{\rm{B}}{\rm{o}}{\rm{u}}{\rm{n}}{\rm{d}}{\rm{a}}{\rm{r}}{\rm{y}}}\,\mathop{\sum }\limits_{\alpha }^{x,y}\,{\gamma }_{\alpha i{\rm{^{\prime} }}(k{\rm{^{\prime} }},l{\rm{^{\prime} }})}{\Gamma }_{\alpha i{\rm{^{\prime} }}(k{\rm{^{\prime} }},l{\rm{^{\prime} }})}\frac{\Delta t}{\Delta \alpha }{P}_{i(k,l)i{\rm{^{\prime} }}(k{\rm{^{\prime} }},l{\rm{^{\prime} }})}{j}_{\alpha i{\rm{^{\prime} }}(k{\rm{^{\prime} }}+{\beta }_{x},l{\rm{^{\prime} }}+{\beta }_{y})}^{m+\frac{1}{2}}\\  &  & -\,\mathop{\sum }\limits_{i{\rm{^{\prime} }},k{\rm{^{\prime} }},l{\rm{^{\prime} }}}^{{\rm{N}}{\rm{o}}\,{\rm{b}}{\rm{o}}{\rm{u}}{\rm{n}}{\rm{d}}{\rm{a}}{\rm{r}}{\rm{y}}}\,\frac{\Delta t}{\Delta x}{P}_{i(k,l)i{\rm{^{\prime} }}(k{\rm{^{\prime} }},l{\rm{^{\prime} }})}({j}_{xi{\rm{^{\prime} }}(k{\rm{^{\prime} }}+\frac{1}{2},l{\rm{^{\prime} }})}^{m+\frac{1}{2}}-{j}_{xi{\rm{^{\prime} }}(k{\rm{^{\prime} }}-\frac{1}{2},l{\rm{^{\prime} }})}^{m+\frac{1}{2}})\\  &  & -\,\mathop{\sum }\limits_{i{\rm{^{\prime} }},k{\rm{^{\prime} }},l{\rm{^{\prime} }}}^{{\rm{N}}{\rm{o}}\,{\rm{b}}{\rm{o}}{\rm{u}}{\rm{n}}{\rm{d}}{\rm{a}}{\rm{r}}{\rm{y}}}\,\frac{\Delta t}{\Delta y}{P}_{i(k,l)i{\rm{^{\prime} }}(k{\rm{^{\prime} }},l{\rm{^{\prime} }})}({j}_{yi{\rm{^{\prime} }}(k{\rm{^{\prime} }},l{\rm{^{\prime} }}+\frac{1}{2})}^{m+\frac{1}{2}}-{j}_{yi{\rm{^{\prime} }}(k{\rm{^{\prime} }},l{\rm{^{\prime} }}-\frac{1}{2})}^{m+\frac{1}{2}}).\end{array}\end{array}$$

We introduced factors ${\gamma }_{xi(k,l)}$ and ${\gamma }_{yi(k,l)}$ to represent the position and the direction (*x* or *y*) of the current density at boundary cell $i(k,l)$. These factors are shown in Table 1, and the position of cell $i(k,l)$ corresponds to boundaries A through D, as shown in Fig. 5. For instance, the case of ${\gamma }_{xi(k,l)}=1$ and ${\gamma }_{yi(k,l)}=0$ means that the input current flows in the *x* direction at boundary A. The factors ${\Gamma }_{xi(k,l)}$ and ${\Gamma }_{yi(k,l)}$ are introduced in Table 1 to consider the shift in the current density by half in Eq. (21). This shift is taken care of by the *β* factor, where ${\beta }_{x}=\frac{1}{2}$ and ${\beta }_{y}=0$ at boundary A, ${\beta }_{x}=-\,\frac{1}{2}$ and ${\beta }_{y}=0$ at boundary B, ${\beta }_{y}=\frac{1}{2}$ and ${\beta }_{x}=0$ at boundary D, and ${\beta }_{y}=-\,\frac{1}{2}$ and ${\beta }_{x}=0$ at boundary C.

**Table 1:** Coefficients *γ*_*x*_, *γ*_*y*_, Γ_*x*_, and Γ_*y*_ for boundary cell *i*(*k*, *l*).

Position	A	B	C	D
*γ*_*x*_	1	−1	0	0
*γ*_*y*_	0	0	−1	1
Γ_*x*_	2	2	0	0
Γ_*y*_	0	0	2	2

Here, A through D represent the boundaries shown in Fig. 5.

We can express the CCE as a matrix equation, as used in Eq. (19):

23 $${{\bf{A}}}_{d}^{T}{{\bf{U}}}_{d}^{m+1}-{{\bf{Z}}}_{d}{{\bf{j}}}_{d}^{m+1}={{\bf{A}}}_{d}^{T}{{\bf{U}}}_{d}^{m}+{{\bf{Z}}}_{d}{{\bf{j}}}_{d}^{m}-{\tilde{{\bf{U}}}}_{d}^{m+1}.$$

Here, matrix **A**_*d*_ is a unit matrix because each cell is not connected to other cells at boundaries. Vector **U**_*d*_ and **j**_*d*_ are the cell potential and the cell current density, respectively. Moreover, **Z**_*d*_ is the time-domain cell impedance matrix expressed as ${{\bf{Z}}}_{d}={\sum }_{\alpha =x,y}\,{\gamma }_{\alpha }\frac{\Delta t}{\Delta \alpha }{\bf{P}}$. We write $\tilde{{\bf{U}}}$ for all terms in the second, third, and fourth summation terms in the right-hand side of Eq. (22) and treat this term as a source term in the CCE.

Next, we can use KCL to derive the connection relation between distributed- and lumped-parameter circuits using the incidence matrix:

24 $$({\gamma }_{x}{{\bf{S}}}_{x}+{\gamma }_{y}{{\bf{S}}}_{y}){{\bf{A}}}_{d}{{\bf{j}}}_{d}^{m+1}+{{\bf{A}}}_{l}{{\bf{I}}}_{l}^{m+1}=-\,{{\bf{A}}}_{s}{{\bf{I}}}_{s}^{m+1}.$$

Here, matrices **S**_*x*_ and **S**_*y*_ are diagonal matrices of size $p\times p$, where *p* is the number of boundaries, and the elements are determined by the cross section of the *β*-th boundary cell (${S}_{x\beta \beta }=\Delta z\Delta y$ and ${S}_{y\beta \beta }=\Delta z\Delta x$).

Finally, we can express the time domain IPE for the coupled lumped- and distributed-parameter circuit by combining Eqs. (19), (23) and (24). We express the variables of the boundary, which are **U**_*l*_, **U**_*d*_, **I**_*l*_, and **j**_*d*_, as follows:

25 $${\bf{U}}=(\begin{array}{c}{{\bf{U}}}_{d}\\ {{\bf{U}}}_{l}\end{array}),\,{\bf{I}}=(\begin{array}{c}{{\bf{I}}}_{l}\\ {{\bf{j}}}_{d}\end{array}).$$

The incidence matrices of lumped- and distributed-parameter circuits are joined horizontally:

26 $${\bf{A}}=({{\bf{A}}}_{l}\,{{\bf{A}}}_{d})$$

The time-domain impedance matrices in both lumped- and distributed-parameter circuits are arranged diagonally:

27 $${\bf{Z}}=(\begin{array}{cc}{{\bf{Z}}}_{l} & {\bf{0}}\\ {\bf{0}} & {{\bf{Z}}}_{d}\end{array})$$

The voltage sources are joined vertically:

28 $${{\bf{E}}}^{m+1}=(\begin{array}{c}{{\bf{V}}}_{s}^{m+1}+{{\bf{V}}}_{s}^{m}\\ {\mathop{{\bf{U}}}\limits^{ \sim }}_{d}^{m+1}\end{array})$$

Finally, we can summarize all of the conditions at boundaries in the same form as Eq. (19):

29 $$(\begin{array}{cc}{{\bf{A}}}^{T} & -{\bf{Z}}\\ {\bf{0}} & {\gamma }_{S}{\bf{A}}\end{array})(\begin{array}{c}{{\bf{U}}}^{m+1}\\ {{\bf{I}}}^{m+1}\end{array})=(\begin{array}{cc}-\epsilon {{\bf{A}}}^{t} & \delta {\bf{Z}}\\ {\bf{0}} & {\bf{0}}\end{array})(\begin{array}{c}{{\bf{U}}}^{m}\\ {{\bf{I}}}^{m}\end{array})+(\begin{array}{c}{{\bf{E}}}^{m+1}\\ -{{\bf{A}}}_{s}{{\bf{I}}}_{s}^{m+1}\end{array})$$

Here, the matrices $\epsilon$ and *δ* are diagonal matrices with the signs chosen according to the *β*-th element of the lumped-parameter circuit and the distributed-parameter circuit, as in Eqs. (30) and (31).

30 $${\epsilon }_{\beta \beta }=\{\begin{array}{cc}-1 & {\rm{f}}{\rm{o}}{\rm{r}}\,{\rm{c}}{\rm{a}}{\rm{p}}{\rm{a}}{\rm{c}}{\rm{i}}{\rm{t}}{\rm{a}}{\rm{n}}{\rm{c}}{\rm{e}}\,{\rm{a}}{\rm{n}}{\rm{d}}\,{\rm{d}}{\rm{i}}{\rm{s}}{\rm{t}}{\rm{r}}{\rm{i}}{\rm{b}}{\rm{u}}{\rm{t}}{\rm{e}}{\rm{d}}\,{\rm{p}}{\rm{a}}{\rm{r}}{\rm{a}}{\rm{m}}{\rm{e}}{\rm{t}}{\rm{e}}{\rm{r}}\,{\rm{c}}{\rm{e}}{\rm{l}}{\rm{l}},\\ 1 & {\rm{f}}{\rm{o}}{\rm{r}}\,{\rm{t}}{\rm{h}}{\rm{e}}\,{\rm{o}}{\rm{t}}{\rm{h}}{\rm{e}}{\rm{r}}{\rm{s}}.\end{array}$$

31 $${\delta }_{\beta \beta }=\{\begin{array}{cc}-1 & {\rm{f}}{\rm{o}}{\rm{r}}\,{\rm{i}}{\rm{n}}{\rm{d}}{\rm{u}}{\rm{c}}{\rm{t}}{\rm{a}}{\rm{n}}{\rm{c}}{\rm{e}},\\ 1 & {\rm{f}}{\rm{o}}{\rm{r}}\,{\rm{t}}{\rm{h}}{\rm{e}}\,{\rm{o}}{\rm{t}}{\rm{h}}{\rm{e}}{\rm{r}}{\rm{s}}.\end{array}$$

The matrix *γ*_*S*_ is also a diagonal matrix to fit the unit of current in KCL and consider the direction of current in the *β*-th distributed-parameter circuit.

32 $${\gamma }_{{S}_{\beta \beta }}=\{\begin{array}{c}1\\ {\gamma }_{x\beta \beta }{S}_{x\beta \beta }+{\gamma }_{y\beta \beta }{S}_{y\beta \beta }\end{array}\,\begin{array}{c}{\rm{f}}{\rm{o}}{\rm{r}}\,{\rm{l}}{\rm{u}}{\rm{m}}{\rm{p}}{\rm{e}}{\rm{d}}\,{\rm{p}}{\rm{a}}{\rm{r}}{\rm{a}}{\rm{m}}{\rm{e}}{\rm{t}}{\rm{e}}{\rm{r}}\,{\rm{e}}{\rm{l}}{\rm{e}}{\rm{m}}{\rm{e}}{\rm{n}}{\rm{t}}{\rm{s}},\\ {\rm{f}}{\rm{o}}{\rm{r}}\,{\rm{d}}{\rm{i}}{\rm{s}}{\rm{t}}{\rm{r}}{\rm{i}}{\rm{b}}{\rm{u}}{\rm{t}}{\rm{e}}{\rm{d}}\,{\rm{p}}{\rm{a}}{\rm{r}}{\rm{a}}{\rm{m}}{\rm{e}}{\rm{t}}{\rm{e}}{\rm{r}}\,{\rm{C}}{\rm{e}}{\rm{l}}{\rm{l}}.\end{array}$$

In the Supplementary Information, we show the calculation results of the time variation of potentials and charge densities in a simple two-layer circuit system, as shown in Fig. 4. Comparing the simulation results of “Supplementary Movie 1.mov” and “Supplementary Movie 2.mov”, the potentials of the bottom layer plane, which are usually used as the ground, largely depend on the position of the upper layer plane.

## Calculation Algorithm of Boundary Conditions to Reduce Calculation Cost

In this section, we explain the appropriate calculation algorithm for an MLP circuit. In the previous algorithm shown in Table 2, we calculated all boundaries using Eq. (29), although the size of the matrix is proportional to the number of cells in the MLP circuit. The calculation is burdensome in the case of a complicated circuit. In the new algorithm, we divide the boundary into two cases, considering that most of the boundaries in the MLP circuit are open. One is the case with a lumped-parameter circuit (W), and the other is the case without a lumped-parameter circuit (WO), as shown in Table 2. We can reduce the size of the matrix in Eq. (29) considerably and obtain a simplified boundary equation in the case of no lumped-parameter circuit.

**Table 2:** Previous and present calculation algorithms for calculation cost reduction.

Location		Previous algorithm^10,11^		Proposed algorithm	
		***U***	***j***_***x***_, ***j***_***y***_	***U***	***j***_***x***_, ***j***_***y***_
Boundary	W	(i) Eq. (29)		(i) Eq. (29)	
	WO			(iii) Eq. (34)	(ii) Eq. (33)
No boundary		(ii) Eq. (16)	(iii) Eqs. (17) and (18)		(vi) Eqs. (17) and (18)

Here, W refers to the boundary with a lumped-parameter circuit, and WO refers to the boundary without a lumped-parameter circuit.

In the case without a lumped-parameter circuit, the KCL at the boundary is derived from Eq. (29):

33 $$({\gamma }_{x}{{\bf{S}}}_{x}+{\gamma }_{y}{{\bf{S}}}_{y}){{\bf{A}}}_{d}{{\bf{j}}}_{d}^{m+1}=0.$$

The current density in the distributed-parameter circuit flowing perpendicular to the boundary is always zero when a lumped-parameter circuit is not connected. As for the condition of the distributed-parameter circuit, the following equation can be obtained by substituting Eq. (33) into Eq. (22):

34 $$\begin{array}{ccc}{U}_{i(k,l)}^{m+1} & = & {U}_{i(k,l)}^{m}\\  &  & +\,\mathop{\sum }\limits_{{i}^{{\rm{^{\prime} }}},{k}^{{\rm{^{\prime} }}},{l}^{{\rm{^{\prime} }}}}^{{\rm{B}}{\rm{o}}{\rm{u}}{\rm{n}}{\rm{d}}{\rm{a}}{\rm{r}}{\rm{y}}({\rm{W}})}\,\mathop{\sum }\limits_{\alpha }^{x,y}\,{\gamma }_{\alpha {i}^{{\rm{^{\prime} }}}({k}^{{\rm{^{\prime} }}},{l}^{{\rm{^{\prime} }}})}\frac{\Delta t}{\Delta \alpha }{P}_{i(k,l){i}^{{\rm{^{\prime} }}}({k}^{{\rm{^{\prime} }}},{l}^{{\rm{^{\prime} }}})}({j}_{\alpha {i}^{{\rm{^{\prime} }}}({k}^{{\rm{^{\prime} }}},{l}^{{\rm{^{\prime} }}})}^{m+1}+{j}_{\alpha {i}^{{\rm{^{\prime} }}}({k}^{{\rm{^{\prime} }}},{l}^{{\rm{^{\prime} }}})}^{m})\\  &  & -\,\mathop{\sum }\limits_{{i}^{{\rm{^{\prime} }}},{k}^{{\rm{^{\prime} }}},{l}^{{\rm{^{\prime} }}}}^{{\rm{B}}{\rm{o}}{\rm{u}}{\rm{n}}{\rm{d}}{\rm{a}}{\rm{r}}{\rm{y}}({\rm{W}},{\rm{W}}{\rm{O}})}\,\mathop{\sum }\limits_{\alpha }^{x,y}\,{\Gamma }_{\alpha {i}^{{\rm{^{\prime} }}}({k}^{{\rm{^{\prime} }}},{l}^{{\rm{^{\prime} }}})}\frac{\Delta t}{\Delta \alpha }{P}_{i(k,l){i}^{{\rm{^{\prime} }}}({k}^{{\rm{^{\prime} }}},{l}^{{\rm{^{\prime} }}})}\,{j}_{\alpha {i}^{{\rm{^{\prime} }}}({k}^{{\rm{^{\prime} }}}+{\beta }_{x},{l}^{{\rm{^{\prime} }}}+{\beta }_{y})}^{m+\frac{1}{2}}\\  &  & -\,\mathop{\sum }\limits_{{i}^{{\rm{^{\prime} }}},{k}^{{\rm{^{\prime} }}},{l}^{{\rm{^{\prime} }}}}^{{\rm{N}}{\rm{o}}\,{\rm{b}}{\rm{o}}{\rm{u}}{\rm{n}}{\rm{d}}{\rm{a}}{\rm{r}}{\rm{y}}}\,\frac{\Delta t}{\Delta x}{P}_{i(k,l){i}^{{\rm{^{\prime} }}}({k}^{{\rm{^{\prime} }}},{l}^{{\rm{^{\prime} }}})}({j}_{x{i}^{{\rm{^{\prime} }}}({k}^{{\rm{^{\prime} }}}+\frac{1}{2},{l}^{{\rm{^{\prime} }}})}^{m+\frac{1}{2}}-{j}_{x{i}^{{\rm{^{\prime} }}}({k}^{{\rm{^{\prime} }}}-\frac{1}{2},{l}^{{\rm{^{\prime} }}})}^{m+\frac{1}{2}})\\  &  & -\,\mathop{\sum }\limits_{{i}^{{\rm{^{\prime} }}},{k}^{{\rm{^{\prime} }}},{l}^{{\rm{^{\prime} }}}}^{{\rm{N}}{\rm{o}}\,{\rm{b}}{\rm{o}}{\rm{u}}{\rm{n}}{\rm{d}}{\rm{a}}{\rm{r}}{\rm{y}}}\,\frac{\Delta t}{\Delta y}{P}_{i(k,l){i}^{{\rm{^{\prime} }}}({k}^{{\rm{^{\prime} }}},{l}^{{\rm{^{\prime} }}})}({j}_{y{i}^{{\rm{^{\prime} }}}({k}^{{\rm{^{\prime} }}},{l}^{{\rm{^{\prime} }}}+\frac{1}{2})}^{m+\frac{1}{2}}-{j}_{y{i}^{{\rm{^{\prime} }}}({k}^{{\rm{^{\prime} }}},{l}^{{\rm{^{\prime} }}}-\frac{1}{2})}^{m+\frac{1}{2}}).\end{array}$$

Here, ${j}_{\alpha i^{\prime} (k^{\prime} ,l^{\prime} )}^{m+1}$ is a known value obtained by Eq. (29). Equation (16) is the same as Eq. (34) when the summation term is divided into boundary and no-boundary terms. In other words, the potential at the boundary where the lumped circuit is not connected can be calculated in the same manner as the no-boundary case when the current density flowing perpendicular to the boundary is set to zero. In this case, we can significantly decrease the size of the boundary matrix in Eq. (29). The calculation algorithms are listed in Table 2.

## Discussion with Numerical Results

We performed numerical calculation using the present method and the corresponding experiment. We then compared the numerical results with the experimental results together with the results calculated by the PEEC method^15,16^. We used the equations and space discretization methods used in the PEEC method. There are differences in the calculation method of boundary conditions and the updating time step between our formulation and the PEEC method. We calculated the boundaries by connecting the lumped-parameter circuit and the transport equations derived from the Maxwell equations, as discussed in the present paper, while the PEEC method converts all finite-volume cells into equivalent circuit models and solves both the boundary and no-boundary parts at the same time. As for time discretization, we used the leapfrog time discretization method, which is the concept of the FDTD method, for updating the potential and the current density^17^, while the PEEC method uses a lumped-parameter circuit simulation algorithm^18,19^.

We consider a two-layer plane circuit with two bends used in the simulation and experiment (see Supplementary Information, Figs 1 and 2). We compare our simulation results with sub-nanosecond time domain reflectometry (TDR) experiments and PEEC method simulation. We can see good agreement between the numerical results of the proposed method and the experimental results (see Supplementary Information, Fig. 3). We perform simulation with both the proposed method and the PEEC method with the same numerical calculation parameters, such as the space and time steps. The proposed method can reproduce the reflected wave with higher accuracy because of the calculation method for boundary conditions and leapfrog time updating.

In addition, we present electromagnetic noise simulation in a simple circuit, which has a micro-strip line configuration, as shown in Fig. 4. The ground noise voltage, which causes external radiation and common mode noise, is generated in various directions according to the position of the signal line^20^ (see Supplementary Information, Movies 1 and 2).

At the moment, the delay time in the scalar and vector potentials in the charge and current densities has been dropped in the numerical calculations. We are currently studying the inclusion of the delay time effect in the present formulation^14^. In addition, multi-layer-plane conductors exist in a uniform medium, and the skin effect is not considered. In order to precisely consider the change of dielectric properties in the conductors and surrounding materials as well as the skin effect, it is necessary to extend our calculation to three dimensions. If three-dimensional calculation is realized, then the propagation speed will change inside and outside of the conductor plane, which means that we can consider the variation of dielectric properties in each plane, as well as the skin effect. We hope to consider these factors in the near future.

This calculation method enables us to visualize transient basic electromagnetic phenomena, such as propagation, reflection, and coupling with other conductors. We can also clarify the relationship between complex circuit shapes and electromagnetic phenomena observed in high-frequency filters, antennas, and meta-materials^21–23^. This calculation method will be important not only in elucidating electromagnetic noise phenomena but also in developing future advanced circuit design.

## Summary

In the present paper, we introduced a method to quantify the transient phenomena of the potential and current density in a multi-layer plane (MLP) circuit. The theoretical equations were obtained from the Maxwell equations. In addition, we used Ohm’s law and continuity equations in order to describe the transport phenomena. In the numerical method, we use the spatial difference method used in the PEEC method and the central difference approximation used in the FDTD method. We also developed a calculation method for the boundary in the MLP and proposed an appropriate calculation algorithm in order to greatly reduce the calculation cost. We applied the present theoretical method for a two-dimensional circuit system and demonstrated the power of the newly developed theory.

## Supplementary information

Supplementary Information

Supplementary Movie1

Supplementary Movie2
