# Supplementary material for: Time-domain Formulation of a Multi-layer Plane Circuit Coupled with Lumped-parameter Circuits using Maxwell Equations
Source: Sci Rep. 2019 Nov 29;9:17891. doi: 10.1038/s41598-019-53288-x (PMC6884524; doi:10.1038/s41598-019-53288-x)
Supplement: Supplementary file 1 — Supplementary Information [file 41598_2019_53288_MOESM1_ESM.pdf]

Time-domain Formulation of a Multi-layer Plane Circuit Coupled with Lumped-parameter Circuits using Maxwell Equations

Souma Jinno[1*], Shuji Kitora[1], Hiroshi Toki[1], and Masayuki Abe[1]

[1]Graduate School of Engineering Science, Osaka University, Toyonaka, Osaka 560-8531, Japan

*Corresponding author : Souma Jinno
*Email : soumajinno117@gmail.com
*Address : Graduate School of Engineering Science, Osaka University, Toyonaka, Osaka 560-8531, Japan

Supplementary Information Guide

1. **Supplementary Figure 1**: A two-layer plane circuit of the same shape as the printed circuit board used in the experiment.
2. **Supplementary Figure 2** : A planar circuit board with a two-bend transmission line used in an experiment to confirm the validity of this calculation technique.
3. **Supplementary Figure 3**: Experimental and numerical results, which are calculated by PEEC and the proposed method.
4. **Supplementary Movie 1**: The time variation of potentials and charge densities in a circuit, which consists of two planes arranged so as to face each other. Here, the upper thin plane is located at the center of bottom plane.
5. **Supplementary Movie 2**: The time variation of potentials and charge densities in a circuit, which consists of two planes arranged so as to face each other. Here, the upper thin plane is located at the edge of the bottom plane.

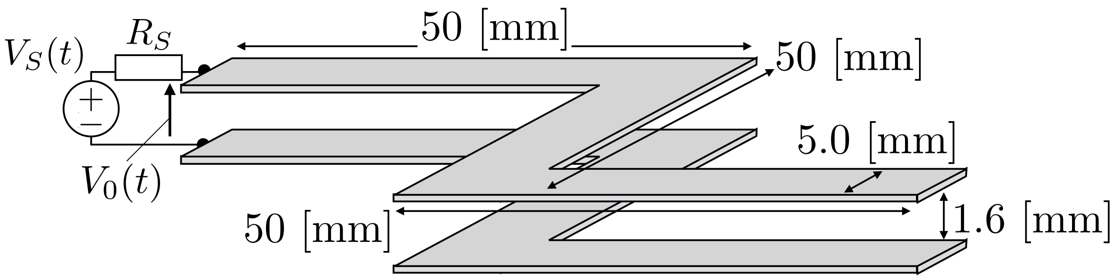

Supplementary Figure 1. This figure shows the circuit configuration used for the simulation. The circuit has two-layer plane conductors, which have a width of 5 mm and bends every 50 mm. These planes have the same size and are separated by 1.6 mm. In the simulation, a lumped voltage source is connected to the left side, and a step voltage $V_S(t)$ of 0.5 V with a rise time of 0.085 ns is input, whereas the right side is open. A time domain reflectometry (TDR) simulation can be performed by observing the time response $V_0(t)$, which is the potential difference between planes on the input side. In the numerical calculation, the conductor plane was divided into cells having a width of 1 mm and a length of 1 mm, and the time division was set to 3.25 ps.

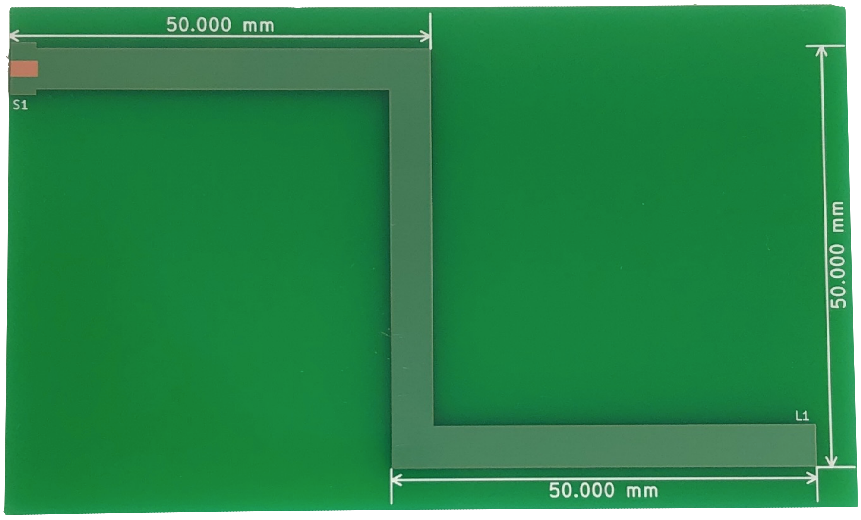

Supplementary Figure 2. This figure shows a top view of the two-layer printed circuit board used for the experiment. The material of the substrate is FR-4, and the solder resist is on the upper surface, except where an SMA connector is mounted at the left side. The parameters for these circuit configurations are the same as those used in the simulation shown in Fig. S1. We use TDR to measure the reflection of the steep step voltage due to the bending of the transmission plane. We use Tektronix DSA8200 and 80E04 to input and measure signals at the left side and use 80A02 to protect experimental equipment from electrostatic discharge. A step voltage was input to the circuit board using an SMA connector and coaxial cable, SMA(P)-50-STF358.

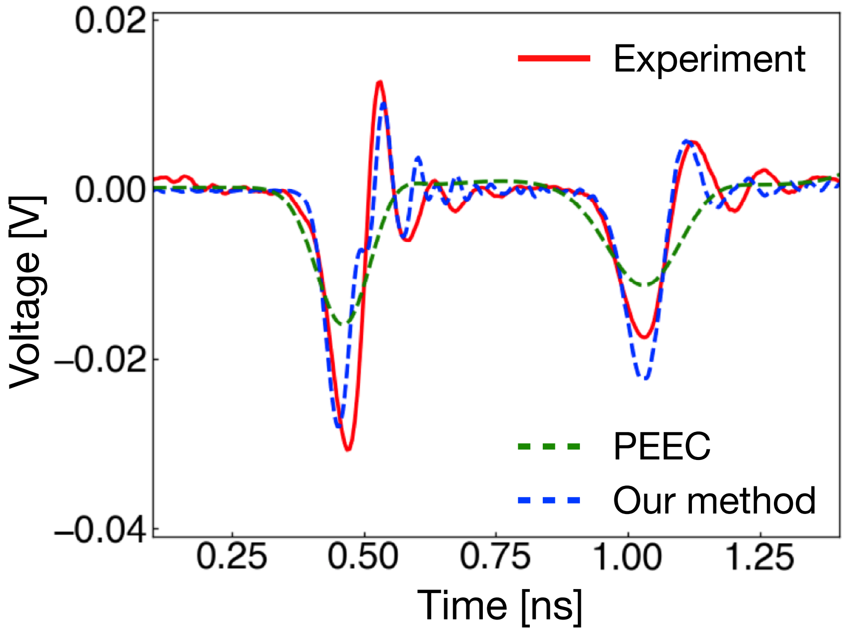

Supplementary Figure 3. This figure shows the experimental results (red solid line) and numerical results, which are calculated by the PEEC method (blue dashed line) and the proposed method (black dashed line). Here, we set relative dielectric ε_r to 3.8 from the propagation speed in the transmission plane of experimental results. Here, we set $V_0(t)$ to 0 V when the step voltage rises by offsetting negative voltage, so 0 V means no reflection, and the potential difference means the reflected wave. We can see a good agreement between the experimental result and the numerical result of our calculation method. The same waveform pattern, which is the reflected wave by the bend, appears twice. The numerical parameters used in the PEEC method and the proposed method are exactly the same. In the PEEC method simulation, we formulated the circuit equation using the sparse tableau method from the equivalent circuit model and solved the equation using the backward Euler method. The proposed method can more precisely reproduce the reflected waveform formed by the shape of the bend.

Supplementary Movie 1. This movie shows the time variation of potentials and charge densities in a simple two-layer plane circuit system as shown in Fig. 4 in the paper, where the upper thin plane is arranged at the middle of the bottom plane. The left side figures represent the results of potentials, and the right side figures represent the charge densities. The squares expressed by the dotted lines represent the reference plane, which is 0 V or 0 mC/m in each layer. Here, a-a′ and b-b′ denote the connection points of lumped-parameter circuits on the power supply side and the load side, respectively. The upper plane has a narrower width of $W_1 = 0.01$ m, and the bottom plane has a wider width of 0.1 m. The length of both planes is L = 0.1 m, and the thickness is $\Delta z = 0.0003$ m. The distance between the planes is H = 0.001 m. The relative permittivity is 2.2. We input the voltage, which is a pulse wave of 10 V in amplitude and 1 ns in rise and fall time, at the left side of the circuit by connecting an independent voltage source that has an internal resistance of $R_S = 50\ \Omega$. We also obtain the impedance matching by connecting appropriate resistance of $R_L = 26\ \Omega$ at the right side. In the numerical calculation, we divide the two planes into small thin square cells of 0.002 m in both width and length. In this case, the signals are dampened in a short time, and a small oscillation continues in the x-direction.

Supplementary Movie 2. This movie shows the time variation of potentials and charge densities in a two-layer plane circuit system, which is modified from the case of a symmetrical arrangement, where the upper plane is shifted toward the edge by 0.04 m from the middle of the bottom plane. The left side figures represent the results of potentials, and the right side figures represent charge densities. The squares expressed by dotted lines represent the reference plane, which is 0 in each layer. Here, a-a′ and b-b′ denote the connection points of lumped-parameter circuits on the power supply side and the load side, respectively. The results show that the signals continue for some time, and some oscillations continue in the bottom plane, not only in the x-direction but also in the y-direction, due to the asymmetrical arrangement.
